# Tectal etiology for irrepressible saccades: a case study in a Rhesus monkey

**DOI:** 10.12688/f1000research.2-85.v2

**Published:** 2013-09-10

**Authors:** James W Gnadt, Christopher T Noto, Jagmeet S Kanwal

**Affiliations:** 1 Department of Physiology and Biophysics, Georgetown University School of Medicine, Washington DC, 20057, USA; 2 Department of Physiology and Biophysics, Howard University College of Medicine, Washington DC, 20059, USA; 3 Department of Neurology, Georgetown University School of Medicine, Washington DC, 20057, USA

**Keywords:** Tectal, saccades, Rhesus monkey, vision, eye movements

## Abstract

Brain circuits controlling eye movements are widely distributed and complex. The etiology of irrepressible square wave saccades is not fully understood and is likely different for different neuropathologies. In a previous study, spontaneously occurring irrepressible saccades were noted after a cerebrovascular accident that damaged the rostral superior colliculus (SC) and its commissure in a Rhesus monkey. Here, we tracked and quantified the development of similar symptoms in a Rhesus monkey caused by a lesion in the rostromedial SC and its commissure. We documented the changes in these saccadic intrusions while the monkey attempted fixation of a target on three consecutive days post-onset. On the first day, eye jerk amplitude was ~10 degrees and the direction was ~30 degrees above the left horizontal meridian. On the second day, the amplitude decreased to 6.5 degrees and the direction shifted towards vertical, ~20 degrees to the left of the vertical meridian. Size, but not direction, of the eye jerks continued to decrease until intrusions dissipated within one month. Histological examination after ~6 months from the first appearance of the intrusions revealed a lesion in the commissure of the SC. Results from this and the previous study confirm the involvement of the commissure of the SC as the common target for triggering this neuropathy. Our data suggest that commissural fibers play an important role in maintaining normal visual stability. Interrupting the commissure between the two superior colliculi causes saccadic intrusions in the form of irrepressible jerking of the eyes, probably by disrupting inhibitory signals transmitted through the commissure. Furthermore, disappearance of the symptoms suggests that inhibitory fields within the SC are plastic and can expand, possibly via inputs from inter-collicular and nigrotectal pathways.

## Introduction

We observe the world by making successive fixations disrupted by periods of eye movements. During the periods of fixation, we gather and integrate relevant visual information about our environment and decide where to look next. However, even during these periods of fixation, our eyes are not completely still. Involuntary microsaccades at regular intervals, 1–2 movements per second, move the eyes by ~0.08–0.5 degrees to, refresh the retinal image and thus preserve normal vision ^1^. Also, macrosaccadic activity of the eye (e.g. square wave jerks) has been observed across a broad age spectrum of normal human subjects ^2– 8^, and possibly plays a role in preventing adaptation of visual neurons during continuous exposure to the same image. Square wave eye jerks observed in the majority of subjects showed small (0.5–3 degrees) saccades, which disrupt fixation and are followed by a second saccade, about 200–500 ms later that return eye gaze to the target. These eye jerks (normal instability) can occur at low rates (far fewer than 12 per minute), in any direction, but are usually present in the horizontal direction. These movements happen infrequently and there remain questions regarding their role and necessity during fixation.

Pathologies of the central nervous system (CNS) can result in abnormal instability of the eye. Eye jerks and other irrepressible breaks in fixation caused by saccadic intrusions, saccade oscillations, and saccade dysmetria have been noted as indicators of CNS disease. For example, the well-known basal ganglia disorder, Parkinson’s disease, presents with many types of saccadic intrusions when a patient attempts to fixate ^9– 11^, task-dependent reaction time impairments ^10, 12, 13^, and saccade dysmetria ^10, 14– 17^. Deep brain stimulation in the subthalmic nucleus (STN) diminishes the frequency of saccadic intrusions ^11^ and improves both reaction time ^12^ and the accuracy of memory-guided saccades ^18^. This is important because we know that the basal ganglia project bilaterally to the superior colliculus (SC) within the tectum via crossed and uncrossed projections of the substantia nigra pars reticulata (SNr) (STN-SNr-SC pathway) ^19, 20^. Via this pathway, the influence of the SNr has been postulated to act as an inhibitory regulator of saccade onset, suggesting that appropriate inhibition of SC neurons by the SNr is a major contributing factor to the stability of the eye during fixation ^11, 20, 21^. Indeed, Carasig ^22^ demonstrated that a lesion made through the tectum and commissure of a monkey’s superior colliculus (SC), presumably disrupting SNr signaling, led to a case of irrepressible saccadic intrusions and square-wave eye jerks of approximately 20 degrees. In particular, they described intrusive eye jerk movements that gradually diminished over the course of about 3 weeks.

In addition to Parkinson’s disease and lesions of the superior colliculus, unilateral lesions of the globus pallidus in Parkinsonian patients ^22^ and a unilateral lesion of the raphe interpositus nucleus (RIP) ^23^ have been shown to cause saccadic intrusions. Pallidotomy caused square wave jerks that varied in direction, though in one case the preponderance of eye jerks were directed contraversive to the lesion ^22^. The lesion of the RIP, which contains saccade-related omnipause neurons (OPNs), caused saccadic oscillation of the eyes with ipsiversive-directed primary saccades, though these oscillations may have been caused by mechanisms secondary to interrupting the pathway to the RIP from the contralateral SC ^23^. More focal lesions of the saccade-related RIP in monkeys, show impairment in saccade metrics, decreased movement duration and peak velocity, but found no notable changes in the ability to fix at a target location ^24^. Here we describe intrusions of the type noted above that were created by a serendipitous lesion. We also provide histological evidence for localizing the lesion primarily to the commissure of the SC in the Rhesus monkey. The data reported here confirm a previous observation of the same etiology ^25^, strengthening the significance of our finding and its interpretation.

## Materials and methods

The study presented here describes data collected from one, seven-year old, female Rhesus monkey ( *Macacca mulatta*) obtained from Stony Brook University (Stony Brook, New York). The animal was instrumented with a scleral search coil and recording chambers directed towards the superior colliculus (SC) for obtaining neurophysiological recording. During a regular recording session using a micro-wire, the animal developed saccadic intrusions. As soon as the monkey began exhibiting eye jerks, we recorded horizontal and vertical eye position under normal room lighting and in the dark while the monkey attempted to fixate on a small LED target located straight ahead. At the end of the recording session, we performed a standard neurological examination of the monkey ^25^ to test for cranial nerve and long-tract motor symptoms. Aside from the oculomotor difficulties, there were no neurological impairments of the limbs, posture, mobility or cranial nerve functions. Further neural recording experiments were suspended and for three days post-discovery, we continued to record eye movements while the monkey was fixating a target in ambient light and in the dark.

All surgical and experimental protocols were approved by the Georgetown University Animal Care and Use Committee (protocol #09-025). The animal was handled in a manner which ameliorated distress or suffering, as suggested in the Weatherall report ^26^, and cared for in accordance with institutional guidelines as put forth by AAALAC and United States federal law.

### General animal care and preparation for experiments

Monkeys are housed individually in grooming contact cages or in groups in customized primate cages of 1.6 or 2.5 m ^3^ with view of colony mates in a large open room. Cages are continuously equipped with swings, mirrors, foraging devices and/or small toys. Animals are provided with daily care and medical maintenance, including a balanced diet of dry food formula, vegetables and fruit. Environmental enrichment for the monkeys included playing of natural sounds, radio or TV and daily handling, mock grooming and socialization by laboratory personnel.

Each monkey is first acclimated to handling and interaction with the human investigators over a period of weeks to months and taught to transfer from the home cage to a portable, enclosed chair, which is wheeled into the laboratory for experiments. Once the monkey becomes acclimated to handling, it undergoes aseptic surgical procedures under general anesthesia monitored by veterinary support staff to prepare them for behavioral training of eye movements during visual psychophysics tasks, and neurophysiological recording.

### Implantation of the head restraint post, scleral coil, and recording cylinders

Animals are prepared for participation in experiments by performing two surgeries. For the first surgery, we implant a head restraining device and one scleral eye coil. With the head secured in a stereotaxic device, a 5 cm midline incision is made in the scalp. Periosteum and muscle is retracted using blunt techniques and the calvarium is freed of soft tissue. A 3 cm stainless steel bar, which fits a head restraining apparatus of the primate chair, is attached vertically to the calvarium using surgical stainless steel screws and a mound of acrylic bone cement (e.g. PALACOS, Heraeus Medical) ^25, 27^. The screws are mounted into small burr holes in the bone and buried in the bone acrylic along with the head post and electrical connectors. A scleral eye coil is implanted on one eye. Briefly, the conjunctiva is cut near the limbus and reflected to expose the sclera. A coil made of three turns of Teflon-insulated wire is sutured to the sclera using 6-0 Vicryl (e.g. polyglactin 910, Ethicon Inc.), and the conjunctiva is sutured back over the coil. The ends of the coil wire are led out of the orbit subdermally to the acrylic cap where they are attached to a small electrical connector. One week post-surgery, we begin a daily task specific training regimen. Once training has proceeded to an acceptable level, generally within a few months, another aseptic surgery is performed to implant an eye coil on the second eye and one or two stainless steel recording cylinder(s) are mounted into the head cap under stereotaxic guidance. The acrylic overlaying the appropriate portion of the skull is removed using dental burrs in a hand-held, dental drill and we make a 15 mm craniotomy. Stainless steel recording cylinders are placed over the craniotomy and cemented into place with acrylic bone cement. The sterile interior of each cylinder is secured with a threaded Teflon cap having a pressure-release vent.

### Behavioral training and neural recording

Using standard behavioral shaping procedures, the animals are trained to fixate and to follow small visual stimuli by rewarding them with a drop of fruit juice from a gravity-fed “straw” for successfully completing each series of eye movements defined by the presentation of the stimuli. Training and experimental procedures generally are performed for 1–3 hours.

Prior to the incident we describe and following full recovery, painless extracellular neuronal recordings in the SC were made using standard electrophysiological methods while our subject tracked a target presented on a video monitor located 24 in front of them using fine wire tungsten microelectrodes (0.5 to 1.2 MOhm, 31 gage, Microprobe, Inc., USA) mounted in a guide tube of stainless steel hypodermic tubing several times a week. Trans-dural penetrations are made by a hydraulic microdrive (FHC, Inc., USA) through the bore of a 21 gauge hypodermic needle mounted in a micropositioner that attaches to the outside of the chronic recording cylinders on the subject's head. Neuronal activity is recorded by a high impedance amplifier system (AMC Systems, Inc., USA) and a laboratory computer that also controls and monitors the behavioral tasks.

### Post-incident data collection and analysis

During post-incident monitoring, we recorded the eye position by means of current induced on the scleral search coil ^28^ that was amplified and offset by a phase detector (Riverbend Instruments, Inc., USA) before being passed to a dedicated PC for data sampling at 1 kHz. To document the eye jerks, we collected approximately 100 seconds of data in both the light and dark on four consecutive days while the monkey fixated a central green LED target (0.14 degrees). A post-hoc script read the binary file and differentiated eye position into velocity, allowing us to easily identify the beginning and end of saccades using a 30 degrees/second threshold. The script wrote a text file containing specific information about each movement (e.g. saccade size and direction, peak eye velocity, movement duration, and other measures), which was later used to quantify the metrics of the observed eye jerks. Eye jerks collected in the light and dark were statistically indistinguishable for each day so we pooled these data.

### Histology

Six months after the monkey first started exhibiting saccadic intrusions, we humanely euthanized it with pentobarbital anesthesia and perfused it through the left cardiac ventricle with saline, sodium nitrate, and heparin solution followed by 4% paraformaldehyde. After the perfusion, the brain was removed from the skull and placed in a buffered 20% glycerin bath for 10 days, then sliced into 40 µm sections using a freezing microtome and mounted onto slides. We mounted every 6 ^th^ section from the level of the anterior commissure to the dorsal column nuclei of the medulla, stained the mounted sections by cresyl violet using standard histological methods (e.g. Nissl staining) to reveal cell bodies, and using a light microscope with an attached high-resolution digital camera created photomicrographs of the tissue to illustrate the extent of the lesion.

## Results

**Figure 1.**
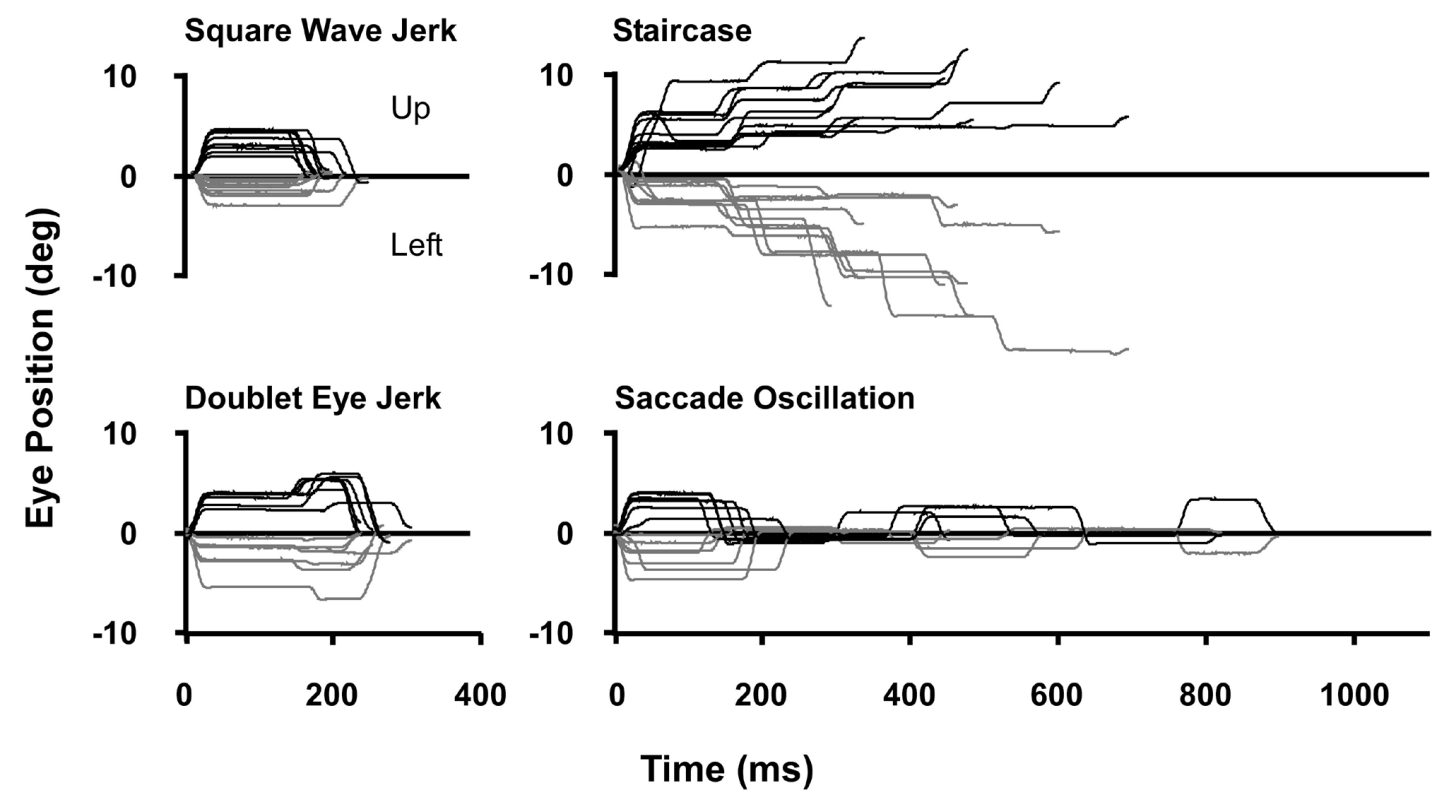
The four types of eye movements evoked by the lesion through the superior colliculus commissure and right rostral-medial superior colliculus. Grey traces show the horizontal component (Left) and black traces the vertical component (Up) of representative irrepressible saccades collected on the third post-discovery day.

Figure 1 illustrates examples of the four fixation deficits observed in this monkey: 1) square wave movements away from the target and back (i.e., square wave jerks), 2) doublets of consecutive eye movements away from the target, 3) multiple, rapid, saccadic “staircase” movements, and 4) saccade oscillations consisting of overshooting saccades across the target. The preponderance of the saccadic intrusions across all days were the square wave eye jerks. The ratio of this type of eye jerk to all other types of saccadic intrusions was nearly 9:1 on the day of discovery and 4:1, 6:1, and 4:1 on successive days. At the first observation of fixation deficits, no doublet eye jerks were made, but we observed a number of staircase saccades. Over the course of the following three days, the number of doublet eye jerks increased until day 3, when we found nearly equal numbers of staircase saccades and doublet eye jerks. Saccades made before the appearance of the syndrome fit well with established main sequence criteria for saccadic quick phases, and so did the fixation-breaking movements of all types of saccadic intrusions. These observations provide convincing evidence for the presence of irrepressible eye movements or saccades that are generated by the brainstem saccade circuit.

### Square wave eye jerk saccades

The frequency of square wave jerk eye movements made by the monkey changed each day. The frequency of movements ranged from 7.8 to 42 per minute with average intersaccadic intervals between 75 ± 11 ms and 143 ± 81 ms. On the first day of the occurrence of intrusions, saccade size averaged 10.1 ± 2.8 degrees and the average direction was 27 ± 15 degrees above the left horizontal meridian. Peak velocities averaged 534 ± 114 degrees per second. On the next day, we observed a significant change. Average saccade size decreased to 7.1 ± 1.2 degrees and the direction moved closer to vertical, 17 ± 8 degrees to the left of the vertical meridian. Velocities decreased to 326 ± 60 degrees per second. On the two succeeding days, saccade amplitude continued to decrease until, on the last day tested, it averaged 4.7 ± 1.8 degrees. Saccade direction did not change further and on the last day was 28 ± 17 degrees to the left of the vertical meridian. Peak velocities averaged 257 ± 83 degrees per second. The decrease in saccade peak velocity was secondary to the decrease in mean saccade size.

### Saccadic intrusions

The square wave eye jerks discussed above consisted of a single saccade away from the fixation point followed by a second saccade returning the eye to the target. The saccadic intrusions we describe here consisted of two (doublets) or multiple (staircases) saccades away from the fixation point followed by a saccade that returned the eye to the target many hundreds of milliseconds later. We considered the doublet and staircase saccades as a single population during the analysis of our data because they seemed to be variations of the same phenomenon that are at different points on a continuum. The general trend across all days was for the monkey to make a large initial saccade with a large vertical component followed by smaller saccades with prominent horizontal components. During their initial occurrence, all saccadic intrusions were directed leftward, and their size averaged <2.5 degrees. They were made in approximately the same direction (~36 degrees above the horizontal meridian). In the days following the lesion, a much larger initial leftward-directed saccade with a large vertical component (4.5–6.5 degrees at 23 degrees to the left of the vertical meridian) was noted. This was followed by smaller, more oblique or horizontally directed saccades. On the first day of its occurrence, staircases of saccades occurred with a one-time maximum of ten consecutive saccades away from the fixation point. Commonly, 3–6 movements occurred during each staircase pattern. On the first day of occurrence of abnormal jerk eye movements, the interval between successive movements averaged 61.5 ± 24.6 ms, an interval about 1/4 that of the normal intrasaccadic latency. On the three following days, we observed a daily increase in the intrasaccadic interval toward normal: first 114 ± 41, then 142 ± 26, and finally 161 ± 80 ms (within the lower range of normal saccade latency, 150–350 ms).

### Saccade oscillations

Saccade oscillations occurred infrequently, but were more frequent in the presence of ambient lighting. Most oscillations occurred as two or three consecutive saccade pairs, within a 400–600 ms period, but in severe instances the oscillation could last for up to 1 second or more, presumably severely disrupting visual perception. The saccades within a given oscillation varied in amplitudes of <10 degrees, tended to be slightly smaller later in the series, and repeatedly overshot to one side of the fixation point and then the other. The initial movement of the oscillations occurred in the same direction as the previously described saccadic intrusions, leftward and upward.

### Lesion location and size

**Figure 2.**
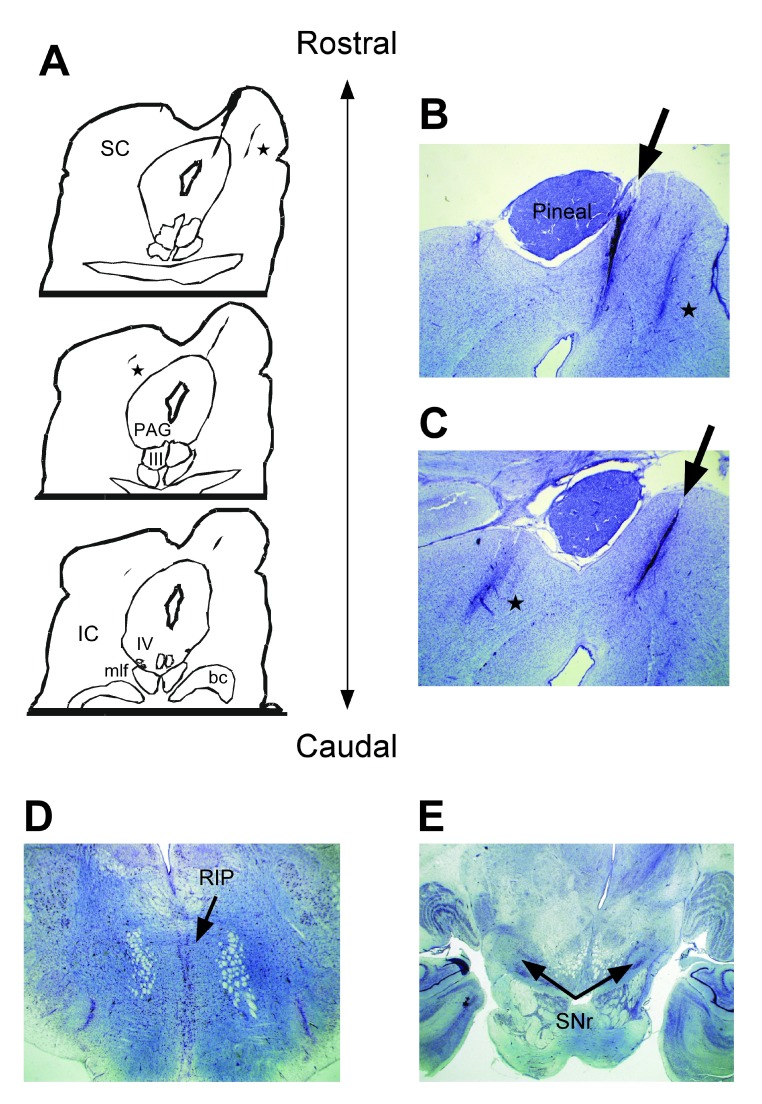
Anatomy of the rostral superior colliculus. Panel **A** shows tracings of the superior colliculus (SC) and other structures in the brainstem relative to the lesion we found bisecting the commissure and the right rostral medial SC. Panels **B** & **C** show photomicrographs taken of the lesion as indicated by arrows. Two other electrode tracts are indicated by asterisks. Panels **D** & **E** show that the RIP and the SNr, respectively, of this monkey remained intact and undisturbed. Abbrevations: SC) superior colliculus, IC) inferior colliculus, PAG) periaqueductal gray, III) oculomotor nerve, IV) trochlear nerve, bc) brachium conjunctivum, RIP) raphe interpositus, SNr) substantia nigra reticulata.

Examination of Nissl-stained brain sections under a bright-field microscope revealed a dorsoventral lesion through the rostral commissure of the SC and rostromedial SC Figure 2A–C illustrate the location and nature of this lesion. Figure 2A shows three consecutive rostral-to-caudal tracings to illustrate the lesion observed at the medial edge of the right SC. This panel reveals essentially a parasagittal “knife cut” lesion at the level of the pineal gland. The lesion ran for approximately 720 µm (3 × 6 × 40 µm sections; one series sampled out of 6) through rostromedial SC and the collicular commissure on the right. Figure 2B and C show photomicrographs of the top two images shown in Figure 2A & C shows the lesion as it extends caudally bisecting the commissure and ~85% of the dorsal-to-ventral extent of the rostral-medial SC. Less extensive gliosis from two other smaller electrode tracts can be also seen in this panel; a lateral one in the right SC, and another that is central and deep within the left SC. These electrode tracts represent penetrations presumably made with either stimulation or recording electrodes at a later date. Figure 2D and E show that two important inhibitory nuclei of the saccadic system, the raphe interpositis pontis (RIP) and the substantia nigra pars reticulata (SNr) remained intact.

The lesion reported here is very similar in size and extent to the one described by Carasig *et al.* ^25^ that caused similar saccadic intrusions. A video of the Carasig subject is available, with permission, as supplemental data. The lesion in the prior study was examined histologically soon (6 weeks) after it occurred, and encompassed roughly 1 mm ^3^ of tissue. In the present case study, the histological assessment of the lesion occurred after a much longer time (6 months), and the long recovery time undoubtedly included the effects of healing that occurred before euthanasia.

A case of irrepressible saccadic intrusions in a Rhesus monkey: data and scripts Irrepressible saccadic intrusions. Data was collected immediately following the inception of the intrusive eye jerks and for the next three consecutive days. Data files contain millisecond by millisecond representations of the eye movement deficits discussed in this manuscript, collected in both the light and dark. Each file contains approximately 100 seconds of data presented here in the Spike2 data format or as a text files.

## Discussion

This paper reports the tectal, specifically collicular, etiology for irrepressible saccades observed after a tungsten wire made a lesion during a routine recording procedure. This wire interrupted the commissure and damaged the rostral-medial part of a Rhesus monkey’s SC. Post lesion, the monkey exhibited four types of saccadic intrusions while trying to fixate on a green LED presented before the animal. The amplitude and direction of these intrusions matched well with what could be expected based on the location of the lesion within the SC motor map. Histological examination of the tectum confirmed that the damaged tissue was located exclusively in the rostromedial part of the SC, which contains neural circuitry for contraversive, upward saccades. No other regions were damaged. Square wave saccadic jerks of the eye were the most frequently displayed abnormal behavior, though we routinely observed doublets of these saccadic movements and even staircases of saccades. Less frequently, the instability in fixation became so great that saccades oscillated about the fixation point for >400 ms. Observations across consecutive days showed a rapid rotation in direction of about 50 degrees from predominately horizontal to predominately vertical, between onset and one day after onset of saccadic intrusions. These intrusions were accompanied by a gradual decrease in eye jerk saccade size.

The data presented in this case study showed generally the same saccadic intrusions in terms of the direction of initial saccade of the eye jerk, from up-right to up-left within 24 hours of onset as reported previously ^25^. Also, the amplitude of the eye jerks decreased over time until about three weeks later when abnormal saccadic intrusions were no longer easily detected.

Our monkey’s saccadic intrusions were also similar to clinical observations of square wave saccadic eye jerks and saccade oscillations in humans. Both human Parkinson’s disease patients ^11^ and our monkeys made eye jerks much greater in size and frequency than considered as normal by many researchers ^3– 8^.

Eye jerks and saccade oscillations are a primary indicator for a wide array of neurodegenerative diseases in addition to Parkinson’s disease (e.g. progressive supranuclear palsy ^9^, multiple sclerosis ^29^, and cerebellar degeneration ^30^). However, two conspicuous differences exist between saccadic intrusions induced by lesions of the SC and those caused by CNS disease. First, the square wave jerks and oscillations made following the SC lesion were oriented obliquely, which is uncommon in CNS degeneration where eye jerks occurred only along one axis, usually horizontal ^5, 30, 31^. Note however that vertical saccadic intrusions have been observed in clinical settings ^31^. A second difference is that the size and frequency of saccadic intrusions made following the SC lesion gradually diminished over about a one-month period. After that time, we observed normal fixation. We speculate that this occurs due to plastic rebalancing of excitatory and inhibitory influences within the tectal, inter-collicular and nigrotectal pathways following this small and discrete neural insult, which like the case of Carasig *et al.* ^25^ involved less than a cubic millimeter of tissue. Saccadic intrusions caused by CNS degeneration never diminish or go away without surgical or drug therapies. For example, deep brain stimulation reduced the frequency of eye jerks caused by Parkinson’s disease ^11^ while administration of diazepam, thiamylal, phenobarbital, and clonazepam were shown to nearly stop saccade oscillations in a patient with cerebellar and brainstem atrophy ^32^. The eye jerks and oscillations, however, return without ongoing intervention. This suggests that the plastic-adaptive mechanisms of the saccade system are also degraded during these types of CNS degeneration, or that the adaptability of the system has been taxed maximally before appearance of symptoms. Therefore, further compensation of the instability of the eye during fixation is no longer possible.

Lesions of the globus pallidus ^22^ or RIP ^23, 24^ also cause square wave jerks and saccadic oscillations, respectively, as well as changes in saccade dynmaics. However, inspection of histology from our subject showed that these structures were intact. The square-wave jerks most likely were caused by unbalanced excitation and inhibition at a focused site in the SC, as illustrated by the preferentially-directed, amplitude-specific saccades generated by our subject following the lesion. This is in contrast to the varied direction of square wave jerks observed by general disinhibition of the SC following unilateral pallidotomy. Doublets and staircases were most likely caused by an unbalanced and prolonged disinhibition of a specific site within the SC motor map. Experimental studies to remove ^19^ or augment ^20^ the modulating inhibitory influence of the nigrotectal system reveals a likely mechanism for the etiology of this site-specific, pathologic disruption in ocular muscle tone. In particular, Liu and Basso ^20^ reveal a crossed nigrotectal inhibition that could be disrupted by transection of fibers transversing the commissure of the SC. In addition, disruption of descending inhibition from the lesioned area of the SC to RIP could also contribute to the observed etiology ^33^.

## Conclusion

We have shown that a lesion through the SC commissure and rostral SC was coincident with the onset of an eye jerk syndrome in monkeys. Current literature describing the role of the commissure and rostral SC, in both monkey and human, states their importance in the inhibition of saccade onset ^11, 12, 20, 21^. In other words, both areas are involved in the permissive side of saccade generation. Presumably by disrupting the balance of excitation and inhibition within a specific region of the rostral-medial SC, motor maps become biased and cause saccades towards the vector space corresponding to the location of the lesion.
