# Supplementary material for: Tectal etiology for irrepressible saccades: a case study in a Rhesus monkey
Source: F1000Res. 2013 Sep 10;2:85. Originally published 2013 Mar 12. [Version 2] doi: 10.12688/f1000research.2-85.v2 (PMC3829126; doi:10.12688/f1000research.2-85.v2)
Supplement: Peer review history

# Peer review: Referee response for version 2

**DOI:** 10.5256/f1000research.1722.r3972

**Published:** 2014-03-06

**Authors:** Michele A Basso

**Affiliations:** 1 UCLA Semel Institute for Neuroscience and Human Behavior, Los Angeles, CA, USA

In my comment on the previous version, I suggested that the authors use superior colliculus instead of tectum. In the "Changes from Version 1", the authors state that they have changed this, but the title still uses tectal.

I have read this submission. I believe that I have an appropriate level of expertise to confirm that it is of an acceptable scientific standard.

# Peer review: Referee response for version 1

**DOI:** 10.5256/f1000research.1166.r962

**Published:** 2013-05-23

**Authors:** Farrel Robinson

**Affiliations:** 1 Department of Biological Structure, University of Washington, Seattle, WA, USA

The title is appropriate and the methods and analysis are appropriate to this article’s conclusions.  

The conclusions are mostly fine. My one comment is that the authors cite reference 23 at least twice to support the statement that lesions of the RIP cause saccadic intrusions. Ref 23 is a report of one clinical case whose brain damage is almost certainly not restricted to RIP and not even very well defined. In contrast, a careful study which specifically damaged the RIP in 3 monkeys reports a result consistent across all 3 animals and very different from that in ref 23 ( Kaneko, 1996, J Neurophysiol. 75:2229-2242.)

The authors clearly present enough data here to support their conclusions.

I have read this submission. I believe that I have an appropriate level of expertise to confirm that it is of an acceptable scientific standard.

# Peer review: Referee response for version 1

**DOI:** 10.5256/f1000research.1166.r850

**Published:** 2013-03-18

**Authors:** Michele A Basso

**Affiliations:** 1 UCLA Semel Institute for Neuroscience and Human Behavior, Los Angeles, CA, USA

This is a straightforward case observation with a clear result and sound interpretation.

I would prefer the authors to use ‘superior colliculus’ instead of ‘tectal’, since the optic tectum generally refers to the structure in non-mammalian species.

Could the oscillations be due to encroachment of the lesion on the pre-tectum? (I know, it probably should be called pre-colliculus). Also, some comparison with the oscillations observed after muscimol injections in SC might be useful.

I have read this submission. I believe that I have an appropriate level of expertise to confirm that it is of an acceptable scientific standard.
